# Longitudinal Follow-Up of Physical Activity During School Recess: Impact of Playground Markings

**DOI:** 10.3389/fpubh.2018.00283

**Published:** 2018-10-04

**Authors:** Georges Baquet, Julien Aucouturier, François Xavier Gamelin, Serge Berthoin

**Affiliations:** University of Lille, University of Artois, University of the Littoral Côte d'Opale, EA 7369 - URePSSS - Unité de Recherche Pluridisciplinaire Sport Santé Société, Lille, France

**Keywords:** children, accelerometry, behavior, multilevel analysis, intervention

## Abstract

To promote physical activity (PA) among children, few studies have reported long-term effects of playground marking during school recess. The aim of this study was to investigate the impact of a playground design on children's recess PA across 12 months and to evaluate the influence of covariates on the intervention effects with accelerometry data. Two hundred and eighty-three children (aged 6–11 years) were selected from 3 elementary schools. Two experimental schools received a recess-based intervention; the third one served as a control group. The design of playgrounds was based on a multicolored zonal design. Children's PA was measured with a uniaxial accelerometer twice a day (morning and afternoon recess) during a 4-day school week. Times spent below and above different PA levels, varying from sedentary (SED, <1.5 METs), light PA (LPA, <4 METs), and from moderate to very high (MVPA, ≥ 4 METs) were calculated before and after 6 and 12 months intervention. A three level (time, pupil, school) multilevel analysis was used to control the intervention effect across time on SED, LPA, and MVPA. The playground intervention was effective after 6 months for LPA (+2.5%, CI 0.65/4.29, *P* < 0.01) and after 12 months for MVPA (+3.1%, CI 0.62/5.54, *P* < 0.01). Moreover, negative non-significant intervention effects were found for SED and LPA. Baseline PA and sex were significant covariates to the contrary of body mass index and age. Playground markings intervention can modify positively long-term school recess total PA.

## Introduction

An insufficient level of physical activity (PA) is a major problem in industrialized countries. Sedentary activity appears early in life (1) and therefore the promotion of physical activity has become necessary in childhood. Habitual PA level of an adult is also partly determined by the level of PA in childhood (2). Faced with growing health problems, including the increased prevalence of overweight and obesity, a consensus has been established for children and adolescents, suggesting 60 min of at least moderate daily PA and incorporating three times a week intense PA (3, 4).

In 2001, Sallis et al. (5) concluded that school environments with high levels of supervision and improvements stimulated girls and boys to be more physically active. Since children spend a substantial time at school, its role in the development of related PA behaviors is very important. Physical Education sessions and recess times are ideal settings to promote PA times because most children attend school and thus can be targeted (6). Habitual PA during recess determines in part the level of PA in children (7), and specific amenities playgrounds allowed a significant increase in habitual physical activity of children (8). These playground markings or additional play equipment allowed to improve total PA and moderate-to-vigorous PA (MVPA) during recess (9, 10). Ridgers et al. (10) have investigated the effect of such a making over time, showing an increase of children's morning and lunch MVPA and vigorous PA (VPA). However, this effect was decreased between 6 and 12 months, highlighting potential confounding variables that influence the intervention effect. Moreover, light PA (LPA) and sedentary activity were not investigated. In children, MVPA and sedentary behavior are independent (11) and then can be influenced by different factors (12). Few data currently exist on correlates of MVPA and sedentary behavior during recess over time. Van Kann et al. (13) have reported that implementation of a multicomponent schoolyard PA intervention did not result in 12 months changes in MVPA. A larger proportion of recess time was spent in light physical activity, which was most likely the result of a shift from sedentary behavior to light physical activity. However, PA was only monitored during the morning recess, which reflects only one of three major PA occasions during a school day, morning, lunch time, and afternoon recess. Further research on recess intervention is needed to examine concerning the effectiveness and feasibility of the effects of interventions in this context on sedentary behavior and PA, PA can be of light, moderate or vigorous intensity.

The purpose of this study was to follow-up the effects of a school-based playground markings intervention on children's recess physical activity levels over 12 months and to highlight factors associated with sedentary behavior and different levels of PA.

## Materials and methods

### Participants

**Figure 1:**
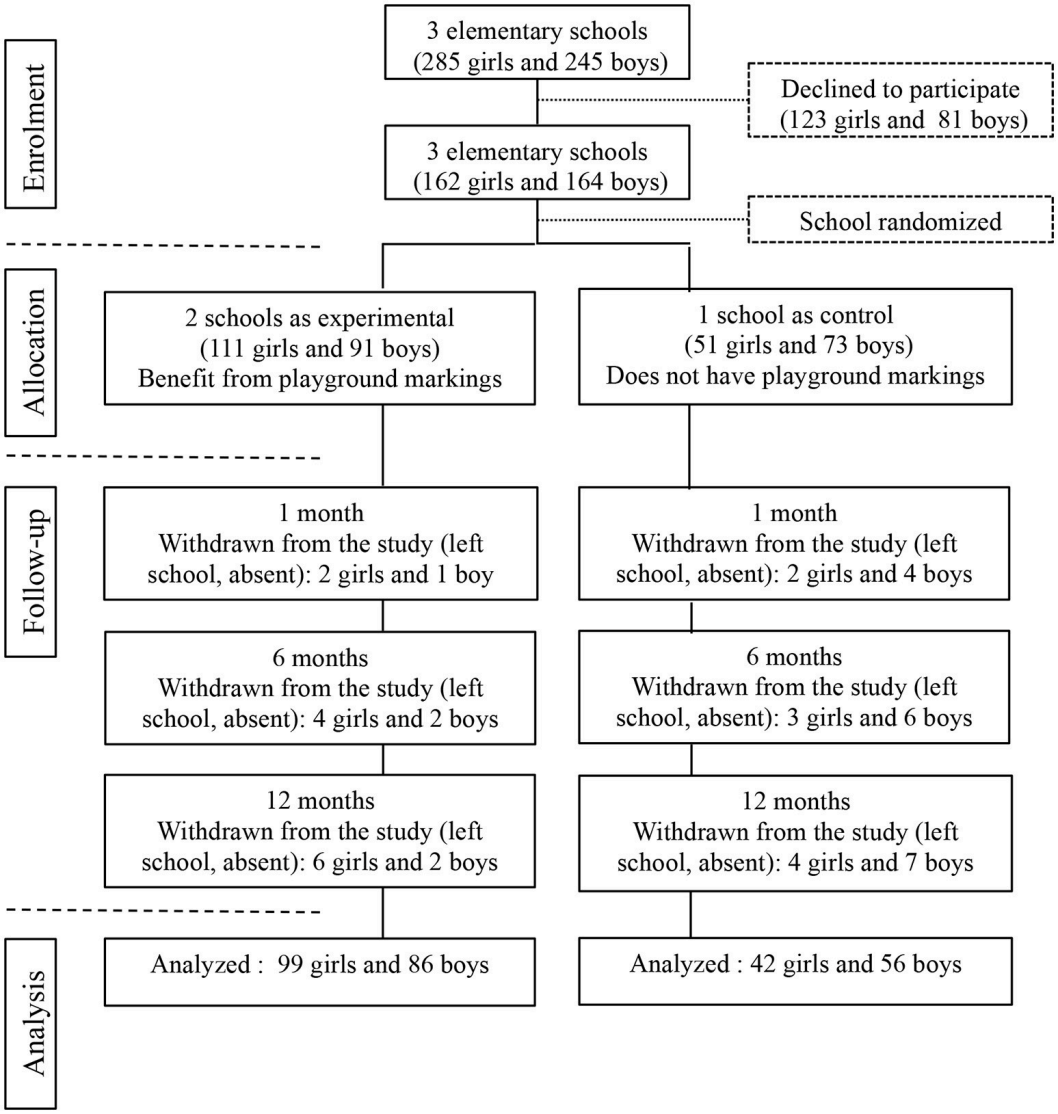
Enrolment, allocation, follow-up, and analysis in the school-based intervention. Measurements were taken at baseline, 1, 6, and 12 months post-intervention.

Three elementary schools located in the same geographical area in the north of France were recruited to participate in the study. There was no ethnic distribution of children. The elementary schools were representative of the Lille suburban area, had similar playground space (around ~1,300 and 1,500 m^2^) and were randomly assigned to experimental and control groups. The flow of children and schools through the study is shown in Figure 1. Three hundred and twenty-six children (162 girls and 164 boys) aged 6–11 years old and their guardians gave informed written consent to participate. The experimental group (EG) included 202 children (111 girls and 91 boys) and the control group (CG) 124 children (51 girls and 73 boys). The study was designed in accordance with ethical standards of the Helsinki Declaration of 2008 and received approval from the “Comité Consultatif de Protection des Personnes en Recherche Biomédicale de Lille.”

### Anthropometric measurements

Height was measured to the nearest 0.1 cm with a wall stadiometer (Vivioz Medical, Paris, France) and body mass was measured to the nearest 0.1 kg with a calibrated electronic balance (Tanita TBF 543, Tanita Inc, Iokyo, Japan). Body Mass Index (BMI) was calculated according to equation: BMI = body mass (kg)/height ^2^ (m^2^). Child weight status was based on BMI percentile cut off points (normal weight: 5%–<85%; overweight: 85% and above) according to WHO (14).

### Physical activity monitoring

Children's PA was assessed with a uniaxial accelerometer (The ActiGraph®, Manufacturing Technologies, Inc., model GT1M), during school recess time (morning, 10–10:15 a.m. and afternoon, 3–3:15 p.m.) only, over 4 school days (Monday, Tuesday, Thursday, Friday). The ActiGraph device facilitates the measurement of human movement (frequency and intensity) over a user-specified time epoch. In this study, the epoch was set at 2 s (15). Accelerometers were distributed in the morning when the children arrived at school and were returned after the afternoon recess period. Data were downloaded for statistical analysis.

### Interventions

The experimental schools received specific playground markings with thermoplastic girdles (Magical Markings, UK), which cost 15,000 Euros per school. The intervention playground environment was based on the sporting playground zonal design (16). This involved a playground division into three specific games (17) and three color-coded areas: (1) a yellow “quiet zone” with non- active games (e.g., chess and drafts), (2) a blue “multi-activity” area for physical fitness and motor skills improvement, and (3) a red 'sports' area (e.g., football, basketball). Children with their teachers were associated to the design of the playground. Fun trails and dens, hopscotch or designs of dragons, clock faces, pirate ship, snakes, or ladders were evenly spaced throughout the playground area. Prior to the intervention, the use of portable play equipment was not allowed by the intervention and control schools. Play equipment (e.g., rackets, balls, huge dies chess, scarfs, hockey sticks…) was provided in the intervention school playground areas by the schools following the redesign (18). Schoolteachers supervised morning and afternoon recess periods.

### Data reduction

Files with missing data were deleted. Times spent below and above different PA levels, varying from sedentary (SED, <1.5 METs, light PA (LPA, <4 METs), moderate PA (MPA, <6METs, to vigorous PA (VPA, 6 ≥METs) and from moderate to very high (MVPA, ≥ 4 METs), were calculated before and after 1, 6, and 12 months intervention. ActiGraph outputs analyzed following the procedures of Trost et al. (19). To compare the time spent in different PA levels between groups, PA time is reported as the percentage of total recess time (morning and afternoon).

### Statistical analysis

Data collected from 43 children (21 girls and 22 boys) who had withdrawn from the study or left the school were rejected. Children who were absent from school on the day of testing or experienced monitor problems were recorded as missing data at that point. Finally, 283 children (141 girls and 142 boys) were retained for the statistical analysis. The experimental group included 185 children (99 girls and 86 boys) and CG 98 children (42 girls and 56 boys). Independent *t*-tests were conducted to examine gender and intervention group differences in baseline variables. All values are expressed as mean ± standard deviation (mean ± SD).

As children's physical activity measurements are not independent of each other in the same environment, a multilevel model was used to take into account this dependency and to determine the effects of the playground intervention (20). To analyze the hierarchical nature of physical activity measurements, a three level (time, pupil, school) multilevel analysis was used to control the intervention effect across time on SED, LPA, MPA, VPA and MVPA. Timing of the follow-up measurement (1, 6, 12 months; level 1), pupils (level 2), and schools (level 3) served as the grouping variables. Potential confounding variables were added to the model as they may influence the effect of intervention. Time (1, 6, 12 months) was level 1 variable and sex, age, baseline physical activity, and BMI group (normal, overweight, obese) were level 2 variables. The intervention term was constructed using a dummy variable, where “0” indicated a control group school, and “1” indicated an intervention school.

Data were analyzed using MLwiN 2.30 software (University of Bristol, UK). In all cases, threshold for significance was set at *p* < 0.05.

## Results

**Figure 2:**
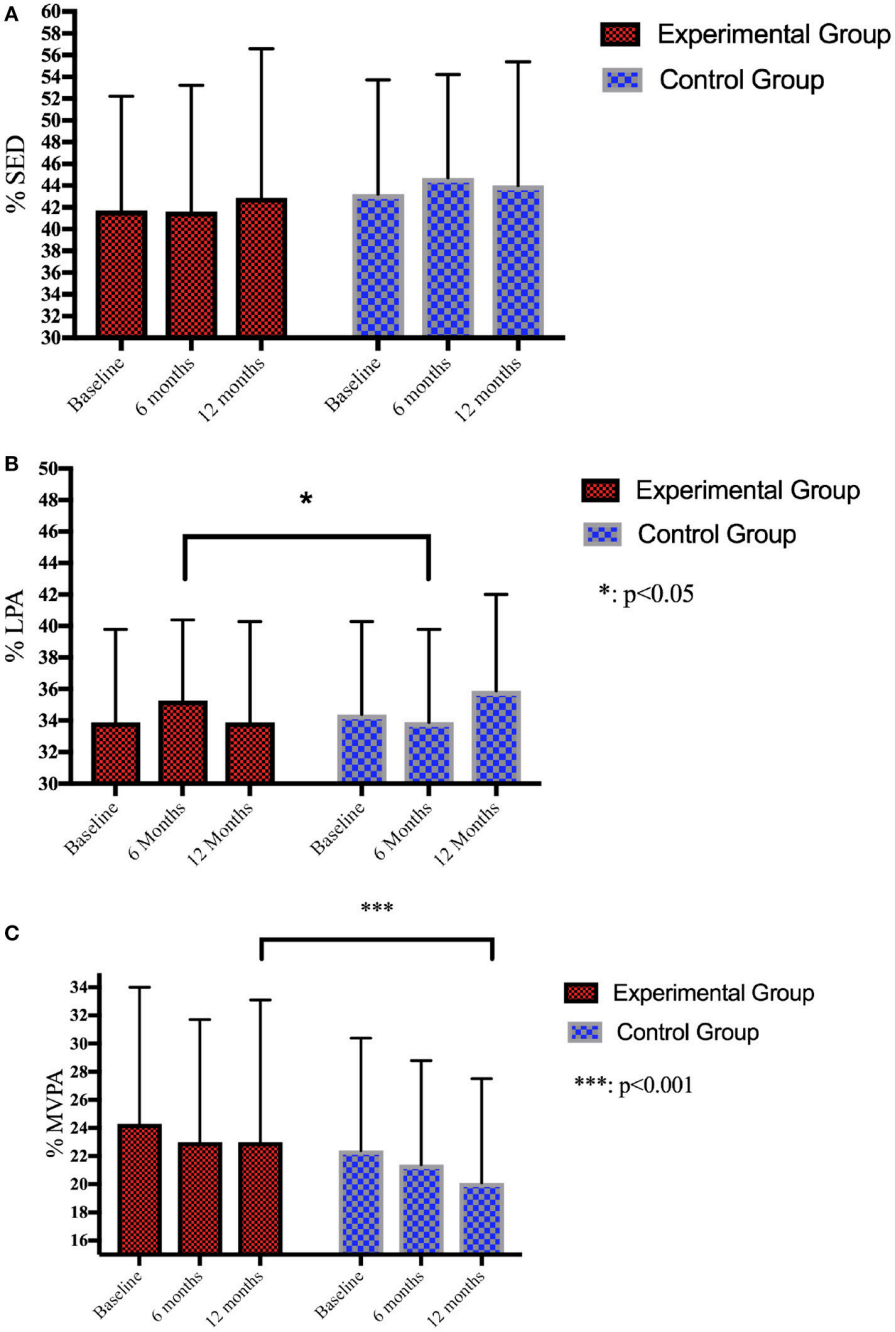
**(A)** Follow-up of SED during recess over 12 months. **(B)** Follow-up of LPA during recess over 12 months. **(C)** Follow-up of MVPA during recess over 12 months.

Age, anthropometric data and baseline physical activity levels of the children are presented in Table 1. Physical activity data during intervention were displayed in Figures 2A–C. The results of the multilevel analysis are reported in Table 2.

**Table 1:** Descriptive baseline and anthropometric and physical activity data at baseline.

	**Boys**		**Girls**	
**Baseline**	**EG (*n* = 86)**	**CG (*n* = 99)**	**EG (*n* = 56)**	**CG (*n* = 42)**
Age (years)	8.5 ± 1.2	8.1 ± 1.8	8.1 ± 1.1	8.1 ± 1.6
Height (m)	1.3 ± 0.1	1.3 ± 0.1	1.28 ± 0.1	1.3 ± 0.1
Body mass (kg)	31.1 ± 7.7	28.3 ± 6.9	28.2 ± 6.5	27.9 ± 6.4
BMI (kg.m^−2^)	17.5 ± 3.0	16.7 ± 2.1	17.0 ± 2.4	16.9 ± 2.0
% SED	35.6 ± 10.2	38.5 ± 10.6	45.0 ± 10.6	44.1 ± 8.6
% LPA	32.2 ± 6.0	33.6 ± 6.2	32.6 ± 5.4	33.1 ± 4.6
% MVPA	32.1 ± 8.9^*^	27.9 ± 8.00	22.4 ± 8.0	22.9 ± 7.6

EG, experimental group; CG, control group; BMI, body mass index; SED, sedentary; LPA, light physical activity; MVPA, moderate to vigorous physical activity;

* *significantly different from CG boys at p < 0.05*.

**Table 2:** Average change in recess physical activity levels (% recess) across two follow-up measurements (6 and 12 months) from baseline in experimental group children compared to control group following a paintings playground intervention.

	**%SED**			**%LPA**			**%MVPA**		
	**β (SE)**	**95%CI**	***p***	**β (SE)**	**95%CI**	***P***	**β (SE)**	**95%CI**	***p***
Baseline PA	**0.23 (0.04)**	**0.15–0.31**	<**0.001**	**0.27 (0.04)**	**0.19–0.35**	<**0.001**	**0.35 (0.04)**	**0.27–0.43**	<**0.001**
Intervention (6 months)	**–**3.12 (1.64)	−6.33–0.09	0.06	**2.47 (0.93)**	**0.65–4.29**	<**0.001**	1.41 (1.13)	−0.80–3.62	0.21
Intervention (12 months)	−1.94 (1.71)	−5.29–1.41	0.26	−1.29 (0.98)	−3.21 to 0,63	0.19	**3.36 (1.18)**	**1.05–5.94**	<**0.001**
Sex (female)	**7.26 (0.88)**	**5.53–8.98**	<**0.001**	−0.34 (0.43)	−1.18 to 0.50	0.42	–**5.44 (0.66)**	–**6.73 to**−**4.14**	<**0.001**
Age	−0.02 (0.02)	−0.06–0.02	0.39	0.003 (0.01)	−0.02–0.02	0.84	0.02 (0.02)	−0.02–0.06	0.74
BMI group (overweight)	−0.53 (0.99)	−2.47–1.41	0.59	0.26 (0.54)	−1.32–1.32	0.63	0.24 (0.74)	−1.21–1.69	0.29
Time (6 months)	**4.12 (1.38)**	**1.41–6.82**	<**0.05**	0.28 (0.77)	−1.23–1.79	0.71	–**5.01 (0.97)**	–**6.91–3.01**	<**0.001**
Time (12 months)	**3.47 (1.44)**	**0.65–6.29**	<**0.05**	**2.55 (0.81)**	**0.96–4.14**	<**0.01**	–**6.59 (1.01)**	–**4.61–3.01**	<**0.001**

*For intervention, time, sex, and Body Mass Index (BMI) group, control group, 1 month, boys and normal weight children are the reference groups, respectively. A positive β value indicates a positive intervention on physical activity (PA) levels of the experimental group compared with the control group during recess over time. The intervention β value represents the difference in physical activity levels for the experimental group against the control group when time, sex, age, BMI group are included in the final model. Bold value represent a significant value*.

### At baseline and at follow-up

At baseline, no significant differences were found on the anthropometric data for the boys and the girls. EG boys engaged in lower levels of MVPA during recess than CG boys (*p* < 0.05) while no significant difference was found between EG and GG in girls for PA levels.

### Intervention effect on change in SB, LPA, and MVPA

Table 2 shows the effect of the intervention on SED, LPA, and MVPA at the 6 and 12 months follow-up measure. A significant positive intervention was found for LPA and MVPA. Children from EG engaged in 3.36% (CI: 1.05–5.94, *p* < 0.001) more MVPA than CG after 12 months of intervention and in 2.47% (CI: 0.65–4.29, *p* < 0.01) more LPA than CG after 6 months of intervention. No significant intervention was found for SED.

Statistical analyses showed that sex was a significant negative variable of LPA and MVPA during the intervention. Boys were engaged in significantly more MVPA (5.44%, CI: 4.14–6.73, *p* < 0.001) while girls spent significantly more time in SED (7.26%, CI: 5.53–8.98, *p* < 0.001).

Body mass index and age were not significant predictors for more or less SB, LPA, and MVPA after intervention.

## Discussion

The aim of the current study was to follow-up the effects of a school-based playground markings intervention on children's recess physical activity levels over 12 months using accelerometry data. The school-based playground markings intervention showed an increase in the time spent in MVPA over time. This result is in accordance with previous studies (10, 18–20). An increase in MVPA is in contrast to VanKann's intervention study (13) that showed positive LPA outcomes but no effect on MVPA. In a review, Ickes et al. (21) reported that a variety of recess interventions has been found to be effective in increasing PA. However, the small number of intervention studies does not allow to establish conclusive effects on children's recess PA (22) and most of them lasted less than 12 months. Moreover, due to the short-term nature of these studies, increases in MVPA may be attributable to a “novelty effect” because the playful aspect of markings arouses children's curiosity. However, after 6 months, we can no longer be considered as a “novelty effect.”

To our knowledge, few studies have investigated sustained effects of school-based intervention on PA (10, 13, 23). Ridgers et al. (10) demonstrated a positive effect on MVPA and vigorous physical activity (VPA), but the PA levels were lower at 12 months compared with 6 months. The present study showed that time was a significant positive predictor of SED and LPA, but MVPA decreased significantly over time. The strongest impact of the intervention was observed at 12 months for MVPA and 6 months for LPA to the contrary of Ridgers et al. (10). Ridgers et al. (23) underlined the influence of confounding variables on the effect intervention (equipment, temperature, play space per child). Seasons can influence the level of physical activity of children (24). Weather conditions are generally linked to lower PA and higher sedentary, as children, in case of a very rainy, snowy or icy day, stay in classrooms. The present experiment began in April and ended 1 year later, with an intermediate measurement in November (6 months). This may explain the greater impact of the intervention at 12 months rather than at 6 months. In Ridger's paper (10), only the PA data from the morning and lunch recess periods were retained, while this current monitored PA during morning and afternoon recess. The lunch break period is longer and then children spent longer time in MVPA (18, 25, 26). Indeed, in this present study, not all children eat at school (27), which can influence not only their PA level, nature of commuting between school and home and after-meal activities (28), but also those who remain in school during lunch break, have more space on the playground to be active (29). The longer recess after lunch break period also allows to better improvement in PA level notably when organizing children's PA with coaches, while the periods of the morning and afternoon are free.

By the way, Vann Kann et al. (13) underlined the importance to make interventions more understandable, especially by involving coaches simultaneously with new play equipment or playground paintings. They implemented a variety of schoolyard PA interventions. The comprehensiveness of PA interventions might be a key to increase MVPA at school in a sustainable way (30). The nature of intervention is also questionable. They identify what type of intervention most affected the changes in recess SB and PA over time. Physical schoolyard interventions decreased time spent in SB and increased, but not significantly, time spent in LPA and MVPA. This approach showed that the more physical environmental stimuli were implemented, the larger the change in SB. However, the playground stimuli in the present study have not decrease SB but significantly increase MVPA. When implementing painting playgrounds or any material to increase PA levels at school, there is a need to understand how children play during recess and what are their expectations regarding this implementation. In this study, the children contributed to the development of the playground by giving their opinion on the type of game they wanted.

To the contrary of Ridger's study (10), age was not a negative predictor of physical activity during recess. Older children were as active as young children. Younger children as older children seem to benefit to the same manner of the playground spaces. However, we cannot identify children's behaviors and the playground spaces they used. Generally, older boys play soccer during recess and, girls and younger boys were engaged in different activities in the remaining playground space. We could not conclude that playground has modified the previous hierarchy. Younger children and girls have certainly benefited from the new playground by accessing more playground spaces. However, playground activities introduced are generally more suited to younger elementary children than older ones. Ridgers et al. (10) conclude that a combination of accelerometry and direct observation to identify children's behaviors and playground spaces they used would give more information. Van Kann et al. (13) have used Global Positioning System devices (GPS) to test whether children were exposed to playground paintings at schoolyard or not. The interest of this device lies in being able to determine the real impact of the playground paintings (decrease of SB and increase in PA) on the real time of exposure and not on the time of the recess. If the goal of this type of intervention was to increase physical activity and to tackle playground issues, GPS could be used in recess studies to determine how changing the playground environment influences activity and playground issues.

## Conclusion

This study demonstrates that a playground markings intervention had a positive effect on MVPA when assessed using accelerometry. However, this intervention did not result in 12-months changes in SED and LPA. There is a need for further studies to consider the real exposure in this environment on children's physical activity levels in combining observation or GPS and accelerometry.

## Author contributions

GB was involved in acquisition, analysis and interpretation of data, drafting, and manuscript writing. JA was involved in analysis and interpretation of data, drafting, and critically revising the manuscript. SB and FXG were involved in the conception and design of the paper and played a role in critically revising and editing the manuscript. All authors read and approved the final manuscript.

### Conflict of interest statement

The authors declare that the research was conducted in the absence of any commercial or financial relationships that could be construed as a potential conflict of interest.
